# Enzymatic Reactions in a Lab-on-Valve System: Cholesterol Evaluations

**DOI:** 10.3390/molecules24162890

**Published:** 2019-08-09

**Authors:** Jucineide S. Barbosa, Marieta L.C. Passos, M. das Graças A. Korn, M. Lúcia M.F.S. Saraiva

**Affiliations:** 1 LAQV, REQUIMTE, Department of Chemical Sciences, Laboratory of Applied Chemistry, Faculty of Pharmacy, Porto University, Rua de Jorge Viterbo Ferreira, 228, 4050-313 Porto, Portugal; 2 Instituto de Química, Departamento de Química Analítica, Campus Universitário de Ondina, Universidade Federal da Bahia, 40170-115 Salvador, Bahia, Brazil; 3 INCT de Energia e Ambiente–Instituto de Química, Campus Universitário de Ondina, Universidade Federal da Bahia, 40170-115 Salvador, Bahia, Brazil

**Keywords:** cholesterol, serum samples, lab-on-valve, automation, enzymatic reaction

## Abstract

The micro sequential injection analysis / lab-on-valve (µSIA-LOV) system is a miniaturized SIA system resulting from the implementation of a lab-on-valve (LOV) atop of the selection valve. It integrates the detection cell and the sample processing channels into the same device, promoting the reduction of reagent consumption and waste generation, the improvement of the versatility, and the reduction of the time of analysis. All of these characteristics are really relevant to the implementation of enzymatic reactions. Additionally, the evaluation of cholesterol in serum samples is widely relevant in clinical diagnosis, since higher values of cholesterol in human blood are actually an important risk factor for cardiovascular problems. An automatic methodology was developed based on the µSIA-LOV system in order to evaluate its advantages in the implementation of enzymatic reactions performed by cholesterol esterase, cholesterol oxidase and peroxidase. Considering these reactions, the developed methodology was also used for the evaluation of cholesterol in human serum samples, showing reliable and accurate results. The developed methodology presented detection and quantification limits of 1.36 and 4.53 mg dL^−1^ and a linear range up to 40 mg dL^−1^. This work confirmed that this µSIA-LOV system is a simple, rapid, versatile, and robust analytical tool for the automatic implementation of enzymatic reactions performed by cholesterol esterase, cholesterol oxidase, and peroxidase. It is also a useful alternative methodology for the routine determinations of cholesterol in real samples, even when compared with other automatic methodologies.

## 1. Introduction

The popularity of biocatalysis has been increasing in the last decades, due to the high specificity and selectivity of enzymes. At the same time, they have been used to substitute some hazardous reagents, and due to their biodegradability, being in accordance with the principles of Green Chemistry. On the other hand, their use can present some drawbacks, such as their dependence on some factors such as temperature, ionic strength, pH and cofactors. Additionally, they can suffer inhibition by some compounds, and their lifetime is limited [1]. In order to overcome these limitations, enzyme has been used in flow-based methodologies. Flow systems allow efficient control of the reaction conditions, maximizing the enzyme activity [1,2], and increasing the reproducibility.

Additionally, using a flow system, the measurements can be done in non-equilibrium conditions increasing the number of analyses per time, and decreasing the amounts of reagents, leading to decreasing costs per analysis, and to a reduction of waste generation. Thus, a large number of enzymatic reactions have been implemented in flow systems, and in particular in sequential injection systems (SIA) [1]. The operation principle of SIA systems is the sequential aspiration of reagent and sample to a holding coil. After that, the product zone is propelled, by the reversal flow, to the detector [3].

The µSIA-LOV [4], whose representation is presented in Section 3.2, is basically a miniaturization of a SIA system, and works on the same SIA principle. The miniaturization results from the implementation of a lab-on-valve (LOV) atop of the selection valve. The LOV integrates the detection cell, leading to an analytical flow path with an internal volume of microliters [4]. Sample processing channels were also integrated into the same device, promoting the proximity between the injection port and the flow cell. Additionally, the possibility of integrating the optical fibers, responsible for the detection, in different positions, allows the implementation of different kinds of detection (absorbance, fluorescence, reflectance) [4], making the system very versatile. The sample processing achieved by reversed flow and by random access provided by the multiposition valve is also very flexible [4]. Thus, this miniaturized system allows, even when compared with SIA, the reduction of reagent consumption (important for enzymatic reactions due to the high reagents costs) and waste generation, improve the versatility of the system and reduce the time of analysis [5]. Additionally, the reduction of sample and reagents volumes promote an improvement in the overlapping of sample and reagents zones, improving the assays repeatability [6]. All of these characteristics are really relevant to the implementation of enzymatic reactions. Additionally, it is also possible to implement the bead injection concept, using different kinds of beads for the sample treatment (preconcentration or matrix removal) [4], or also for enzymes immobilization [1,4]. Thus these previous characteristics turn a µSIA-LOV system into an interesting and challenging alternative to implement enzymatic reactions. Thus, some works about the implementation of enzymatic reactions in µSIA-LOV systems have been reported [6,7,8,9,10,11,12].

**Scheme 1:**
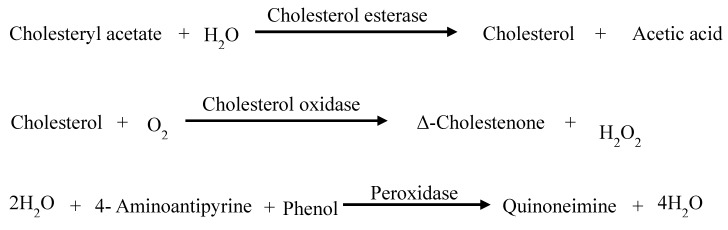
Enzymatic reactions used for the quantification of cholesterol.

In this work it is intended to evaluate, for the first time, the µSIA-LOV potentialities in the implementation of three reactions catalyzed by cholesterol oxidase, cholesterol esterase and peroxidase, following described in Scheme 1.

In these reactions, cholesterol ester is hydrolyzed by the cholesterol esterase, to free cholesterol. This free cholesterol is then oxidized by cholesterol oxidase to cholest-4-en-3-one simultaneously with the reduction of O_2_ to hydrogen peroxide. The hydrogen peroxide can react with different chromogenic indicator systems such as ABTS, 3-dimethylaminobenzoic acid (DMAB)-aminoantipyrine (AAP), or 3,5-dichloro-2-hydroxybenzene sulfonic acid (DCHBS)-AAP to be detected spectrophotometrically [13]. In this work, phenol with 4-AAP was selected as the chromogenic system, and the measurement of the formed quinoneimine is proportional to the total concentration of cholesterol [14,15].

Simultaneously with the evaluation of the potentialities of the implementation of the enzymatic reactions promoted by cholesterol oxidase, cholesterol esterase and peroxidase in a µSIA-LOV system, it is also intended to use the resulted automatic methodology to evaluate total cholesterol levels in real samples.

For adults, cholesterol levels less than 200 mg dL^−1^ are desirable. High levels of cholesterol in the blood (hypercholesterolemia) resulted from a non-balanced diet [16], a sedentary lifestyle, and from some genetic factors [16], and all of this can result in cholesterol accumulation in the arterial walls, leading to atherosclerosis (hardening, thinning and chronic inflammation) [17]. Patients with these kinds of problems have an increased risk of stroke [18], ischemic heart disease [19,20] and peripheral vascular disease [21]. Thus, the evaluation of the cholesterol levels in the blood is essential in order to identify the risk of illness in the general population. The development of enzymatic methodologies with this objective has already been reported both in batch [22,23,24,25,26,27,28] and flow analysis mode [13,29,30,31,32,33,34,35,36] (Table 1).

Despite the fact that some methodologies for the enzymatic determinations of cholesterol have already been developed based on flow analysis, from the best of our knowledge, this is the first time that a µSIA-LOV system is used with this goal.

Thus, it is intended to combine the study of the potentialities of the use of µSIA-LOV for the implementation of enzymatic reactions promoted by cholesterol esterase, cholesterol oxidase and peroxidase, with a development of an automatic, robust, reliable, economic and fast methodology for the quantification of cholesterol in serum samples.

## 2. Results and Discussion

In this work, the objective is to implement some enzymatic reactions promoted by cholesterol esterase, cholesterol oxidase and peroxidase, in a µSIA-LOV system and at the same time to use this developed methodology for the quantification of total cholesterol in human serum samples.

### 2.1. Preliminary Studies 

Before the µSIA-LOV system optimization, and since the cholesterol has low solubility in aqueous solutions, the effect of different concentrations of Triton X-100 in the solubilization of this compound were studied. Concentrations in a range between 1.25 and 10% were tested. It was verified that the lowest concentration of Triton X-100 that allows the solubilization of cholesterol was 1.5%. Thus this was the chosen concentration to be used for the preparation of standard solutions of cholesterol acetate.

### 2.2. µSIA-LOV System Optimization

**Figure 1:**
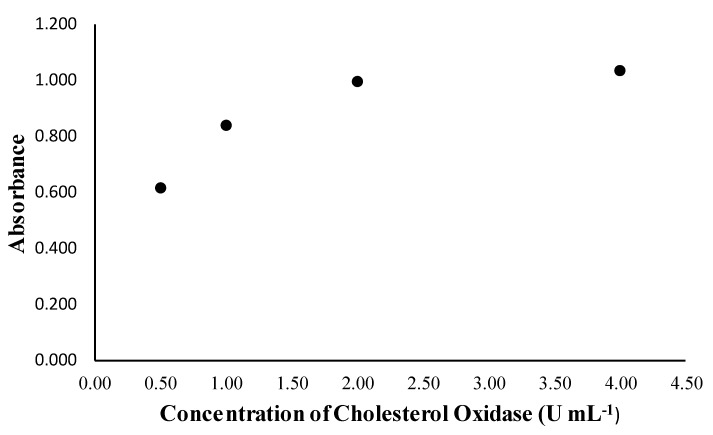
Effect of cholesterol oxidase in the analytical signals.

In order to obtain the best performance with the developed µSIA-LOV system, parameters such as reagent concentrations and volumes, reaction time and other physical factors of the system, were studied. The optimization started by the evaluation of reagents concentrations, in order to certify that any reagent is a limiting reagent for the enzymatic reactions. Regarding the chromogenic reagent, composed by peroxidase enzyme, 4-aminoantipyrine (4-AAP) and phenol, the study started with the concentration of peroxidase. Concentrations between 18 and 146 U·mL^−1^ were tested. Despite the increase of the analytical signals with the increasing concentration of enzyme, the concentration of 36.5 U·mL^−1^ was chosen as a compromise between the sensitivity and the low consumption of the enzyme. The chosen concentration of 4-AAP was 0.10% in a tested interval between 0.02 and 2.00%. The sensitivity was increasing until this concentration (around 91% compared with the concentration of 0.02%), remaining stable for higher concentrations. The concentration of phenol was established in 6% due to its solubility and the intention to spend the least required amount of this toxic compound. For the enzymes, it was considered a concentration of 5.00 U·mL^−1^ for the cholesterol esterase and 2.00 U·mL^−1^ for the cholesterol oxidase (Figure 1) after testing concentrations between 2.00 and 12.5 U·mL^−1^, and 0.50 and 4.00 U·mL^−1^, respectively.

They were the lowest concentrations that lead to the highest sensitivities (around 62 and 24% higher when compared with concentrations of 2.00 and 0.050 U·mL^−1^, for cholesterol oxidase and cholesterol esterase, respectively). The established volumes were 20 µL for the chromogenic reagent and 10 µL for the sample and for the enzymes. However, it was verified that dividing each aliquot into two parts and intercalating them, the mixture was improved, avoiding the formation of double peaks which occurred with the aspiration of the entire aliquots. It happens due to the proximity of the sample injection port to the detection cell. It promotes the use of reduced volumes and this reduction promotes the increase of the overlapping reagents zones [4]. Not only the aliquots division, but also the reagents order of aspiration is widely related to the interdispersion of the solutions inside the system. Thus, the effect of three different orders was studied. The first one was chromogenic reagent (CR)/cholesterol oxidase (CO)/cholesterol esterase (CE)/sample/CE/sample/CO/CR, the second was CR/CO/sample/CO/CE/sample/CE/CR, and the third was CR/CE/CO/sample/CO/CE/CR. The first order was the most efficient, since it leads to a higher sensitivity (around 1%, and 120% higher than with order 2 and order 3, respectively). The flow rate also intervenes in the interdispersion of the reagents, and at the same time influences the time of reaction. This last parameter can also be adjusted using a stopped-flow period, promoting the contact of enzymes, sample and chromogenic reagent, without the dispersion effect. Thus, it was verified that using a flow rate of 1 mL min^−1^ and a stopped-flow period of 6 min, the sensitivity increased around 48% when compared with the use of 5 min period. In the opposite, it was decreased around 15% when compared with a stopped flow period of 10 min, but at the same time, it promoted an increase of 40% in the determination frequency. The flow rate of 1 mL min^−1^ and a stopped-flow period of 6 min were selected as a compromise between sensitivity and the time spent per analysis. The determination frequency is usually higher when a µSIA-LOV system is used, due to the short course of the sample, and the reactants between injection ports and detection cell [4].

**Figure 2:**
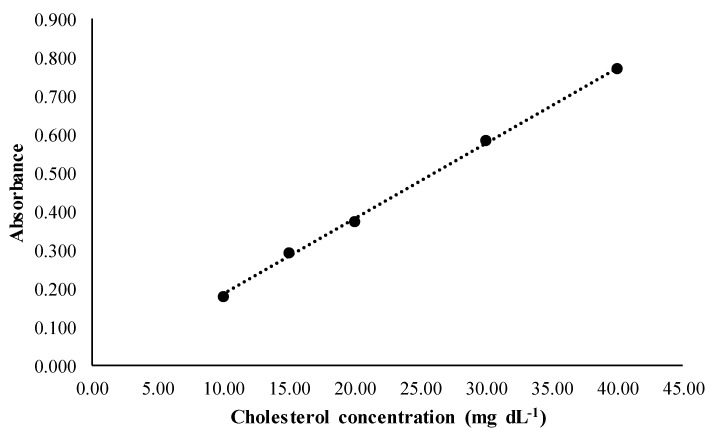
Calibration curve using cholesterol acetate standard solutions.

After the establishment of the previous conditions for the developed µSIA-LOV system, there was performed a calibration curve using cholesterol acetate standard solutions. This calibration curve (Figure 2) is represented by the equation AU = (1.96 ± 0.12) × 10^−2^ Conc. (mg·dL^−1^) − (1.13 ± 2.99) × 10^−2^ (with confidence limits of 95%, for the intercept and the slope) with an R of 0.9995.

The detection and quantification limits are the concentrations obtained for the intercept, plus three and ten times Sy/x, respectively [37]. Thus, the obtained detection and quantification limits were 1.36 and 4.53 mg dL^−1^, respectively.

A relative standard deviation (RSD) of 3.6% was obtained using 10 consecutive injections of 20 mg dL^−1^ of cholesteryl acetate standard solution, and this proved the repeatability of the developed methodology.

Taking into account the time needed to complete an analytical cycle (including the stopped-flow period and the aspiration and propulsion periods) a determination frequency of around 8 determinations h^−1^ was obtained.

### 2.3. Evaluation of Total Cholesterol Concentrations in Serum Samples Using the Developed µSIA-LOV Methodology

In order to attest the efficiency of the developed µSIA-LOV methodology in the quantification of total cholesterol in real samples, some reference samples of human serum (ABX Penta N Control) and some real human serum samples were selected and evaluated. All the samples were diluted in order to obtain different concentrations included in the linear concentration range from the calibration curve. The obtained results were presented in Table 2.

The developed methodology showed to be accurate since the obtained errors between the reference and the obtained concentrations for the total cholesterol in the samples were lower than 5.5%.

Additionally, the absence of statistical differences between real and obtained results was verified, since according to the t-test [37], the tabulated value of 2.16 was higher than the obtained t value of −0.82 (for 95% of confidence level), showing the accuracy of the results.

### 2.4. Comparison between the Developed Methodology and Other Flow-based Methodologies Used for Cholesterol Evaluation 

As expected, the developed methodology, since it is a flow-based methodology, presents some advantages when compared with the batch procedures, namely promoting the reduction of sample and reagents consumption. The proposed methodology also presents some other advantages when compared with other automatic methodologies based on flow analysis (referred in Table 1). When compared with the methodology that refers to the development of a microfluidic chip [36], it presents a lower detection limit and lower sample consumption. In addition, the chip-based flow injection system was more laborious, since it needs the fabrication of functionalized carbon nanotubes working, silver reference and platinum counter electrode layers on the chip.

When compared with the asymmetrical flow field-flow fractionation (AF4) methodology [35], our manifold was much simpler, and the sample consumption was also smaller (50%).

Comparing with the multi-commutated flow procedure [34], the developed methodology allows a reduction in the detection limit, and at the same time, the sample preparation is more “green”, since it does not use isopropanol as a solvent.

The mono-segmented flow analysis [33], as it is supposed by the principle of this methodology, uses air bubbles between samples. This forces the removal of the air from the stream before detection, turning the process more complex and the methodology more irreproducible. Additionally, the consumption of reagents is considerably higher, since with this methodology the reagents are propelled continuously (a mixture of cholate, Triton X-100, phenol and buffer is used as the carrier, and a mixture of three enzymes and 4-aminoantipyrine, as a reagent). The same situation of the continuous propulsion of the reagents happens with flow injection analysis-based methodologies [13,29,30,32]. Additionally, the FIA methodologies proposed by Situmorang et al. [32], Fernandez-Romero et al. [29], and Krug et al. [31] use isopropanol (an organic toxic solvent) in the sample preparation. In four [13,29,31,32] of the five referred FIA methodologies, the sample volumes are higher (4, 7, 9, and 5 times, respectively) than the proposed methodology. Two [13,31] of the previously referred FIA methodologies use the ABTS instead of 4-aminoantipyrine and phenol for the quantification of hydrogen peroxide resulting from the enzymatic reactions. Despite the fact that the ABTS were apparently less harmful than the phenol, it presents some drawbacks, since it can inhibit the peroxidase enzyme [31], or can suffer auto-oxidation in the presence of samples prepared in Triton X-100 and isopropanol [13]. The preparation of the peroxidase electrode referred in one of these FIA methodologies [30] is also laborious, in opposition to the easy handled developed methodology.

## 3. Materials and Methods 

### 3.1. Reagents and Solutions

High purity water (Millipore, Danvers, MA, USA) with a specific conductivity < 0.1 µS cm^−1^ and analytical reagent grade were used for the preparation of all solutions.

A 0.1 mol·L^−1^ phosphate buffer solution with a pH of 7.0 was used as a carrier and also for the preparation of enzyme solutions. This buffer solution was prepared by mixing appropriate amounts of 0.20 mol·L^−1^ Na_2_HPO_4_ (Sigma Aldrich St. Louis, MO, USA) and 0.20 mol·L^−1^ NaH_2_PO_4_ (Sigma Aldrich) solutions, and diluting them twice with water. 

A 2.00 U·mL^−1^ solution of cholesterol oxidase (from *E. coli*) solution was prepared by the dissolution of the solid enzyme (Sigma Aldrich EC 1.1.3.6, 40 U·mg^−1^ of solid) in a buffer solution.

A 5.00 U·mL^−1^ solution of cholesterol esterase (from a porcine pancreas) solution was prepared by a dissolution of the solid enzyme (Sigma Aldrich EC 3.1.1.13, 35 U·mg^−1^ of solid) in 10 mmol·L^−1^ sodium cholate (Sigma) previously prepared in a buffer solution. The sodium cholate acts as a cholesterol esterase activator [24,33].

A 36.5 U·mL^−1^ peroxidase solution was also prepared by the dissolution of an appropriate amount of solid enzyme (Sigma Aldrich EC1.11.1.7, type I, 146 U·mg^−1^ of solid) in a buffer solution. This solution of the enzyme was used in the preparation of the chromogenic reagent, as well as a 6% phenol (Merck) solution, and a 0.10% 4-aminoanthipyrine (4-AAP) (Sigma Aldrich) solution. The mixture of this solution was done with the proportion of 1:0.75:1 (phenol:4-AAP:peroxidase).

In order to prepare the standard solutions of cholesterol ester, and since it has low solubility in water [24], Triton X-100 was used as a solvent [38]. A 100 mg·dL^−1^ of cholesteryl acetate solution was prepared in Triton X-100 1.5% (*v*/*v*). For that, 5 mg of cholesteryl acetate were added and stirred in 75 µL of Triton X-100 (Sigma Life Science), previously heated until 72 °C. After the complete dissolution, 3 mL of water also previously heated were added, and the temperature and the stirring were kept for 30 min. Then, more 1.5 mL of hot water was added, and the stirring and the temperature were kept for more 30 min. The resulted solution was cooled keeping the stirring and finally, the volume of 5 mL was completed with water at room temperature. Using this final standard solution, a different solution with different concentrations were prepared by appropriate dilutions.

A 1.5% Triton X-100 was also prepared according to the previously described procedure in order to be used for the blank assays.

For the evaluation of total cholesterol, certified samples of serum ABX Penta N Control from Horiba Medical, and real human serum samples were used.

### 3.2. Apparatus

**Figure 3:**
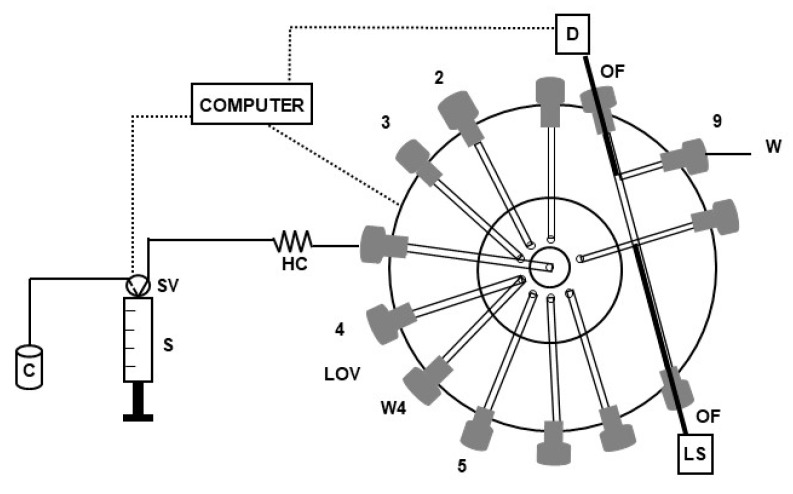
Developed µSIA-LOV system for the implementation of the enzymatic reactions used for the total cholesterol quantification. S, syringe; SV, solenoid valve; C, carrier (Phosphate buffer, pH 7.0); LOV, lab-on-valve; HC, holding coil (with 2 m length and a figures of eight configuration); D, spectrophotometric detector; OF, optical fiber; LS, light source; 2, chromogenic reagent; 3, cholesterol oxidase; 4, sample/standard solution; 5 cholesterol esterase; W, waste; W4, waste from port.

The developed methodology was based in a µSIA-lab-on-valve (LOV) system. This manifold (Figure 3) was composed by a multi-syringe pump model Multi-burette 4S from Crison (Barcelona, Spain) equipped with a syringe of 2.5 mL and a 10-port selection valve (Valco, Vici C25-3180EMH, Houston, USA), where a customized LOV was placed.

The control system was made using a homemade program written in VisualBasic language. A computer with an OOIBase32TM software version 2.0.6.5 of Ocean Optics was used to record the analytical signals.

All of the components of the system were connected using a 0.8 mm i.d. PTFE tubing (Idex).

For the detection, a spectrophotometer USB4000 (from Ocean Optics Inc., Dunedin, FL, USA), a light source (a tungsten halogen lamp, LS-1-LL, from Ocean Optics Inc.) and a pair of optic fibers with 600 µm of core diameter, that connect the light source with the flow cell (integrated in the LOV) and that with the spectrophotometric detector, were used.

### 3.3. Micro Sequential Injection Procedure

The total cholesterol determinations based on the use of enzymatic reaction were performed using a µSIA-LOV. Before the start of the analytical cycle it was necessary to prime the system, filling the central tube with the carrier solution (phosphate buffer solution at pH of 7.0), and the lateral tubes of the selection valve with the reagents. The tubes from positions 2, 3, 4, and 5 were filled with the chromogenic reagent, cholesterol oxidase, sample/standard solutions of cholesteryl acetate and cholesterol esterase, respectively. After this preparation, the analytical cycle (Table 3) was performed by the aspiration of 10 µL of chromogenic reagents (step 1), 5 µL of cholesterol oxidase (step 2), 5 µL of cholesterol esterase (step 3) and 5 µL of sample (step 4). Then, the aspiration of 5 µL of cholesterol esterase (step 5), 5 µL of sample (step 6), 5 µL of cholesterol oxidase (step 7) and 10 µL of chromogenic reagent (step 8), was repeated. After the aspiration of all of these aliquots of reagents, the flow was stopped, the reagents remaining in the holding coil for a period of 6 min (step 9), in order to guarantee enough contact between the enzymes, the sample and the chromogenic reagent. Then, by a flow reversal, the fluids were propelled to the detection cell (a channel between the optic fibers, placed into the LOV) where the absorbance values were measured at 500 nm, and finally, this mixture was sent to the waste (step 10).

### 3.4. Comparison Method

The accuracy of the obtained results with the developed methodology was evaluated in two ways. The first one was the use of commercial reference serum samples. The obtained results for these samples were compared with their theoretical concentration values. The second one was to use real samples of human serum, and compare the obtained concentration values with those obtained in a certified laboratory of clinical analysis, that uses a perfectly implemented methodology based on an enzymatic assay with spectrophotometric detection. These values are presented in Table 2, in the Section 2.3.

## 4. Conclusions

Concluding, a simple automatic methodology using a µSIA-LOV system was developed. It showed to be a useful tool for the application of enzymatic reactions promoted by cholesterol oxidase, cholesterol esterase, and peroxidase enzymes, and also for the evaluation of cholesterol in human serum samples. These results showed to be reliable and accurate, confirming the usefulness of this methodology for the implementation of enzymatic reactions, and at the same time its utility in the clinical field. The automation and the miniaturization leads to the reproducibility improvement, the reduction of reagents consumption, and waste generation. These advantages are relevant even when compared with other automatic methodologies based on the flow analysis. This showed that the developed methodology is in agreement with the principles of green analytical chemistry. Thus, it was verified that the developed µSIA-LOV methodology is a great alternative for the implementation of reactions catalyzed by cholesterol esterase, cholesterol oxidase and peroxidase and for the routine determinations of cholesterol.

## Figures and Tables

**Table 1:** Enzymatic methodologies based on conventional batch and flow-based procedures for the determination of cholesterol levels.

Mode	Methodology	Enzymes	Matrix	Analytes	Detection Mode	Sample Treatment	Linear Range	Detection Limit	Reference
Batch		CO, CE, POD	Serum	Total cholesterol	Amperometry	Dilution with ethanol and triton X-100	100–400 mg·dL^−1^	n.a.	[22]
		CO, CE, POD	Serum	Total cholesterol	Chemiluminescence	Dilution with phosphate buffer solution	n.a.	n.a.	[23]
		CO, CE, POD	Serum	Free and total cholesterol	Chemiluminescence	Dilution with triton X-100	0.4–40 mg·dL^−1^	0.2 mg·dL^−1^	[24]
		CO	-	Free cholesterol	Voltammetry	Dilution with isopropanol and triton X-100	0.2–60.0 nmol·L^−1^	0.05 nmol·L^−1^	[25]
		CO, CE	Serum	Total cholesterol	Amperometry	Dilution with triton X-100	10–700 mg·dL^−1^	0.1 mg·dL^−1^	[26]
		CO, CE	Serum	Total cholesterol	UV-Vis spectrophotometry	Dilution with triton X-100	10–100 µmol·L^−1^	2.9 µmol·L^−1^	[27]
		CO, CE	-	Total cholesterol	Voltammetry	Dilution with isopropanol and triton X-100	5–5000 µg·mL^−1^	3.0 µg·mL^−1^	[28]
Flow analysis	FIA	CO, CE, POD	-	Total cholesterol	UV-Vis spectrophotometry and fluorimetry	Dilution with isopropanol and triton X-100	0.02–0.20 g·L^−1^ 0.005–0.05 g·L^−1^	0.0020 g·L^−1^ 0.0004 g·L^−1^	[29]
	FIA	CO, CE, POD	Serum	Free and total cholesterol	Potentiometry	Dilution with triton X-100	Up to around 10^−3^ mol·L^−1^	3.0 × 10^−3^ mol·L^−1^	[30]
	FIA	CO, CE, POD	Serum	Free and total cholesterol	UV-Vis spectrophotometry	Dilution with isopropanol and triton X-100	0.5–0.8 mmol·L^−1^	n. a.	[31]
	FIA	CO, CE, POD	Serum	Total cholesterol	UV-Vis spectrophotometry	Dilution with triton X-100	0.11–0.86 mmol·L^−1^	n. a.	[13]
	FIA	CO, CE, POD	Serum	Total cholesterol	Potentiometry	Dilution with isopropanol and triton X-100	0.05–3.0 mmol·L^−1^	0.01 mmol·L^−1^	[32]
	MFA	CO, CE, POD	Serum	Total cholesterol	UV-Vis spectrophotometry	Dilution with phenol and triton X-100	Up to 10.3 mmol·L^−1^	n. a.	[33]
	MCFIA	CO, CE, POD	Serum	Total cholesterol	Chemiluminescence	Dilution with isopropanol and triton X-100	25–125 mg·L^−1^	3.7 mg·L^−1^	[34]
	AF4-PFRD	CO, CE, POD	Serum	Cholesterol and triglycerides	UV-Vis spectrophotometry	-	10–250 mg·dL^−1^	n. a	[35]
	FIA microfluidic chip	CO	-	Free cholesterol	Amperometry	Dilution with triton X-100	50–400 mg·dL^−1^	10 mg·dL^−1^	[36]

CO, cholesterol oxidase; CE, cholesterol esterase; POD, peroxidase; FIA, flow injection analysis; MFA, mono-segmented flow analysis; MCFIA, multi-commutated flow injection analysis; AF4-PFRD, Asymmetrical flow field-flow fractionation with on-line, dual post-fractionation reaction detection; n. a., not available.

**Table 2:** Obtained results for the determination of total cholesterol in reference samples of human serum, using the proposed µSIA-LOV methodology.

Kind of Sample	Sample	Standard Concentrations (mg·dL^−1^)	Calculated Concentrations (mg·dL^−1^)	Error (%)
Reference samples	1	109	113.9 ± 0.7	4.5
	2	109	105.5 ± 0.6	−3.2
	3	109	108.6 ± 1.5	−0.3
	4	104	109.3 ± 0.2	5.1
	5	104	105.0 ± 0.0	1.0
Real samples	6	223	228.1 ± 0.1	2.3
	7	283	283.3 ± 1.3	0.1
	8	279	263.6 ± 3.7	−5.5
	9	272	269.2 ± 1.8	−1.0
	10	225	214.4 ± 0.2	−4.7
	11	272	257.4 ± 2.0	−5.4
	12	279	274.7 ± 3.3	−1.5
	13	211	213.5 ± 0.5	1.2
	14	283	292.3 ± 1.1	3.3

**Table 3:** Analytical cycle for the evaluation of total cholesterol.

Step	Position	Volume (µL)	Time (s)	Flow Rate (mL·min^−1^)	Direction	Event
1	2	10	4	0.15	Aspiration	Chromogenic reagent
2	3	5	2	0.15	Aspiration	Cholesterol oxidase
3	5	5	2	0.15	Aspiration	Cholesterol esterase
4	4	5	2	0.15	Aspiration	Sample
5	5	5	2	0.15	Aspiration	Cholesterol esterase
6	4	5	2	0.15	Aspiration	Sample
7	3	5	2	0.15	Aspiration	Cholesterol oxidase
8	2	10	4	0.15	Aspiration	Chromogenic reagent
9	9	-	360	0	Stopped flow	Stopped flow in the holding coil
10	9	10,000	75	1.25	Propulsion	Propulsion to the detector
